# Modulatory Effects of Dabigatran on PAR-1 Activity and Viability in Adipose-Derived Mesenchymal Stem Cells

**DOI:** 10.3390/ijms27093783

**Published:** 2026-04-24

**Authors:** Emre Kubat, Özer Aylin Gürpınar, Tayfun Özdem

**Affiliations:** 1 Department of Cardiovascular Surgery, Özel İskenderun Gelişim Hospital, 31200 Hatay, Türkiye; 2 Department of Biology, Faculty of Science, Hacettepe University, 06800 Ankara, Türkiye; gurpinar@hacettepe.edu.tr; 3 Department of Cardiovascular Surgery, Gulhane Training and Research Hospital, University of Health Sciences, 06010 Ankara, Türkiye; tayfunozdem@yahoo.com

**Keywords:** dabigatran, NOACs, ADMSs, PAR-1 signaling, cell viability

## Abstract

Protease-activated receptor-1 (PAR-1) is a key regulator of mesenchymal stem cell (MSC) migration and tissue integration. Dabigatran, a direct thrombin inhibitor widely used as a non-vitamin K oral anticoagulant (NOAC), may affect PAR-1-mediated signaling pathways. This study investigated the effects of dabigatran on cell viability, apoptosis, and PAR-1 activity in adipose-derived MSCs (ADMSCs) in vitro. ADMSCs were exposed to five concentrations of dabigatran etexilate with thrombin activation. Cell viability was assessed using the MTT assay, apoptosis and morphological changes were evaluated via acridine orange/propidium iodide staining, and PAR-1 expression was analyzed by immunofluorescence. Results showed that high dabigatran concentration significantly reduced cell viability and induced apoptotic morphological changes. In contrast, lower, non-cytotoxic concentrations preserved normal fibroblastic morphology and maintained cell viability while reducing PAR-1 surface expression compared with thrombin-activated controls. These findings indicate that dabigatran at non-cytotoxic doses can modulate PAR-1 activity without compromising ADMSC survival. In conclusion, dabigatran influences MSC-related cellular functions beyond its anticoagulant properties.

## 1. Introduction

Vitamin K antagonists have been used as oral anticoagulants for decades in the treatment and prevention of thromboembolic events [1]. In recent years, NOACs have become widely used to reduce cardiovascular morbidity and mortality [2,3]. Among these agents, dabigatran is an oral direct thrombin inhibitor with rapid, effective, and predictable anticoagulant activity [4].

Under physiological conditions, MSCs are mobilized in response to tissue or organ injury and migrate toward the damaged area [5,6]. MSC migration is regulated through the binding of growth factors and cytokines to surface receptors, which initiate signaling pathways [7,8,9]. PARs, which are expressed on the surface of MSCs, are also known to play a role in MSC migration [10].

However, MSCs administered exogenously for therapeutic purposes have several limitations, including insufficient migration to the target tissue, inadequate tissue adhesion and integration, challenges in maintaining cell viability, and limited cell proliferation [11]. One of the primary goals of cell-based therapies is to overcome these limitations and enhance therapeutic efficacy at the site of application. Under normal conditions, the maintenance of PAR-1 activation, which is responsible for MSC migration, depends on the presence of thrombin; therefore, dabigatran, as a direct thrombin inhibitor, may reduce this effect and negatively influence MSC migration. On the other hand, dabigatran, which is frequently used in cardiovascular diseases, exerts its anticoagulant effect through PAR-1-related mechanisms, and our previous in vitro studies have demonstrated its cytotoxic effects on cells [12]. To the best of our knowledge, limited studies have investigated the effects of dabigatran on PAR-1 activity in mesenchymal stem cells, and direct evidence in this specific context remains scarce no study to date has directly investigated the effect of dabigatran on PAR-1 receptor activity on MSCs. Therefore, the present in vitro study aimed to investigate the effects of dabigatran on cell viability and PAR-1 activity in adipose tissue derived MSCs (ADMSCs).

## 2. Results

### 2.1. Cell Viability and Morphology

**Figure 1:**
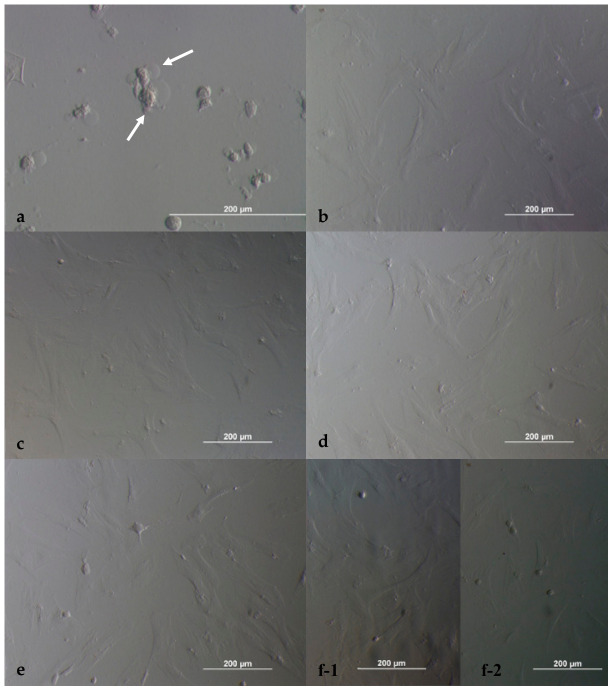
Morphological appearance of ADMSCs exposed to dabigatran at 24 h of incubation. Dilution I (**a**); dilution II (**b**); dilution III (**c**); dilution IV (**d**); dilution V (**e**); and controls (**f**) ((**f-1**): (-) control; (**f-2**): (+) control). Magnification 20 × 10 (**a**) and 10 × 10 (**b**–**f**); scale bar: 200 µm. White arrows indicate the rounded and degenerated cells.

Cell viability results are presented in Table 1. At 24 h, the highest cytotoxic effect was observed in the 24 µM dabigatran group, which demonstrated a statistically significant difference compared with the control groups (*p* < 0.05). No statistically significant differences were observed between the remaining dilutions and the control groups. Morphological evaluation demonstrated that cells treated with 24 µM dabigatran exhibited degenerative, round-shaped morphology (Figure 1a) with nuclear condensation, whereas cells treated with lower concentrations and control groups maintained a healthy fibroblastic morphology (Figure 1b–f).

**Figure 2:**
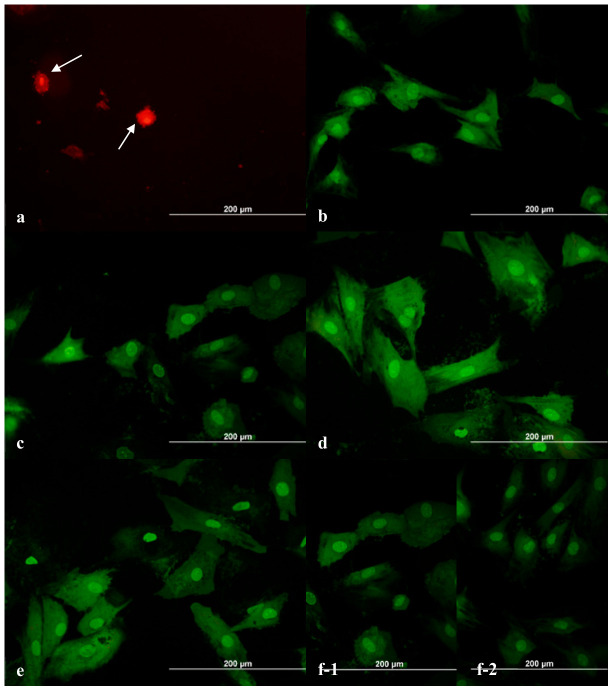
Photomicrographs of AO/PI staining of ADMSCs exposed to dabigatran after 24 h of incubation. Dilution I (**a**), dilution II (**b**), dilution III (**c**), dilution IV (**d**), dilution V (**e**), and controls (**f**) ((**f-1**): (-) control; (**f-2**): (+) control). Magnification 20 × 10; scale bar: 200 µm. White arrows indicate round apoptotic cells and membrane blebbing.

AO/PI staining revealed that cells in the highest concentration group were completely apoptotic (Figure 2a), whereas lower concentrations showed low apoptotic ratios comparable to control groups (Figure 2b–f). The ratio of apoptotic cells at 24 h in each dilution and control groups was given in Table 2. Cells treated with the highest concentration of dabigatran exhibited increased membrane permeability and nuclear changes indicative of cell death. While these features are consistent with apoptosis, the possibility of necrotic or late apoptotic processes cannot be excluded based solely on AO/PI staining.

### 2.2. PAR-1 Staining

**Figure 3:**
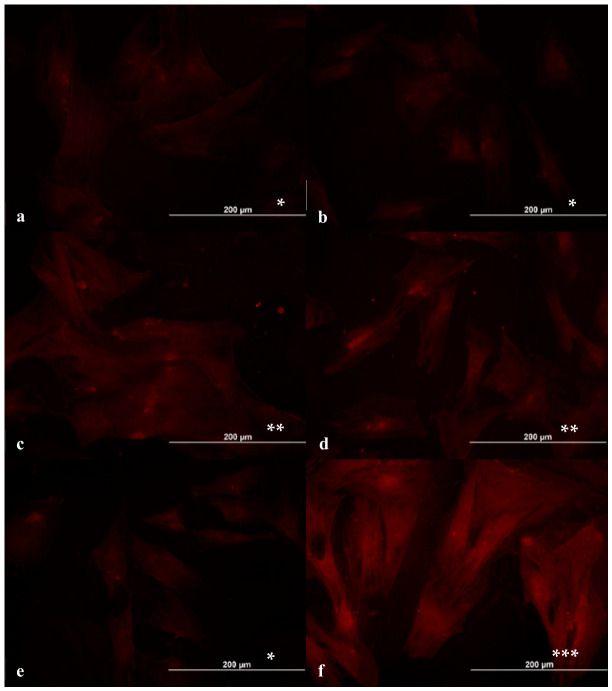
PAR-1 staining of ADMSCs exposed to dabigatran at 24 h of incubation. Dilution II (**a**); dilution III (**b**); dilution IV (**c**); dilution V (**d**); (-) control (**e**); and (+) control (**f**). Magnification 20 × 10; scale bar: 200 µm. (* weak, ** moderate, and *** intense staining).

PAR-1 expression was stronger in thrombin-activated cells than non-activated cells and (-) control groups. Non-cytotoxic concentrations of dabigatran (12 µM—Figure 3a and 6 µM—Figure 3b) showed weak PAR-1 staining similar to the negative control groups. Lower concentrations (3 µM—Figure 3c and 0.75 µM—Figure 3d) demonstrated moderate surface staining, whereas the positive control groups exhibited intense staining on both the cell surface and cytoplasm (Figure 3e). The ratio of PAR-1 (+) cells at 24 h in each dilution and control groups is also given in Table 3. These findings indicate that non-cytotoxic concentrations of dabigatran, a thrombin inhibitor, exhibit a controlled, concentration-dependent suppression of PAR-1 activity.

One limitation of this study is that apoptosis and PAR-1 activity were evaluated using a semi-quantitative morphological counting method based on a fixed number of cells within a defined area. Therefore, the data were expressed as percentages and were not subjected to statistical analysis, which may limit the interpretability and generalizability of the findings.

## 3. Discussion

Myocardial infarction results in the loss of approximately 1 billion cardiomyocytes and 2–3 billion myocardial cells, particularly endothelial cells [13]. MSCs enhance neovascularization and contribute to cardiac regeneration through differentiation and paracrine mechanisms [14]. ADMSCs may differentiate into endothelial cells during neovascularization and contribute to cardiac regeneration [15]. Various studies have also demonstrated that ADMSCs support cardiomyocyte regeneration [16]. Additionally, they activate critical signaling pathways involved in cardiac repair following myocardial infarction [17]. Overall, these studies primarily focused on the effects of MSCs alone, factors secreted by them, or supportive matrices on cardiac regeneration [18]. However, studies investigating the factors that regulate MSC migration after systemic administration and the underlying mechanisms remain limited.

When cardiac injury occurs, growth factors and cytokines bind to receptors on the surface of MSCs and initiate various intracellular signaling pathways that regulate cell migration [19]. Platelet-derived growth factor (PDGF)-BB, PDGF-AB, epidermal growth factor (EGF), heparin-binding EGF-like growth factor (HB-EGF), transforming growth factor-β (TGF-β), insulin-like growth factor-I (IGF-I), hepatocyte growth factor (HGF), fibroblast growth factor-2 (FGF-2), and thrombin—either alone or in combination—affect MSC migration. In certain conditions, thrombin alone selectively enhances MSC migration [19].

Protease-activated receptors expressed on the surface of MSCs are also known to be involved in MSC migration [20]. Protease-activated receptors comprise four subtypes—PAR-1, PAR-2, PAR-3, and PAR-4—which are encoded by different genes [21]. These receptors possess protease-sensitive N-terminal domains that activate intracellular signaling pathways by modifying the extracellular surface of G protein-coupled receptors, thereby regulating MSC migration [21]. Proteolytic enzymes such as trypsin and matrix metalloproteinases (MMPs) play a role in PAR activation [22]. PAR-1 receptors expressed on MSCs are predominantly activated by MMP-1 [23].

Activation of PAR-1 by MMP-1 triggers MAP kinase signaling pathways via associated G proteins, leading to cytoskeletal reorganization and promoting MSC migration and integration into target tissues. In contrast, damage to or inhibition of these receptors suppresses cell migration [23]. Mesenchymal stem cells also express thrombin receptors, and thrombin binding to its receptor enhances fibronectin secretion. The resulting increase in fibronectin subsequently activates PAR-1 receptors, which trigger intracellular ERK1/2 and NF-κB signaling pathways, ultimately promoting MSC migration. Under normal conditions, MSC migration is therefore dependent on the presence of thrombin. The observed reduction in PAR-1 expression at non-cytotoxic dabigatran concentrations may be explained by the inhibition of thrombin-mediated receptor activation. Thrombin cleaves and activates PAR-1, initiating downstream signaling cascades such as ERK1/2 and NF-κB pathways, which are known to regulate cytoskeletal organization and cell migration. By inhibiting thrombin activity, dabigatran may indirectly suppress these signaling pathways, leading to reduced receptor activation and altered cellular responses. This mechanism may partially explain the preserved viability alongside reduced PAR-1 expression observed in our study.

PAR-1 receptors are also expressed on platelets where they are activated by thrombin and play a key role in the coagulation process [24,25]. This mechanism is particularly relevant in anticoagulant therapies for cardiovascular diseases. Dabigatran is an oral direct thrombin inhibitor with rapid, effective, and predictable anticoagulant activity [4]. Dabigatran, a widely used oral anticoagulant, has been increasingly recognized for its cytotoxic effects in various studies. In particular, a study utilizing a gastric epithelial cell line reported that dabigatran induces cytotoxicity via an increase in mitochondrial reactive oxygen species (ROS), aligning with its known gastrointestinal side effects. The literature also documents dabigatran’s cytotoxic effects in primary chondrocytes [26], as well as in osteoblasts and osteoclasts [27]. In our previous investigations, we evaluated the cytotoxic effects of dabigatran on the L929 fibroblast cell line [12,28], demonstrating that it exerts significant cytotoxic and apoptotic effects at doses above the critical threshold. The concentrations of dabigatran used in this study were selected based on previous in vitro studies. Reported peak plasma concentrations of dabigatran in clinical settings are typically in the low micromolar range. Therefore, the lower concentrations tested in this study may be considered within a clinically relevant range, whereas higher concentrations may exceed physiological levels and should be interpreted with caution.

While the cytotoxic potential of dabigatran has been explored in multiple cell types, no studies to date have examined its effects in mesenchymal stem cells (MSCs). Furthermore, the impact of dabigatran on PAR-1 activity within MSCs remains unexplored. Nevertheless, considering that MSCs express both thrombin and PAR-1 receptors, the effect of dabigatran, a thrombin inhibitor, on these cells warrants attention. As previously noted, thrombin inhibition prevents PAR-1 activation, thereby impairing MSC migration.

In the present study, our results showed that PAR-1 expression was weak on the surface of non-activated cells (Figure 3a–e) and stronger in thrombin activated cells (Figure 3f). Non-cytotoxic concentrations of dabigatran (12 µM—Figure 3a and 6 µM—Figure 3b) showed weak PAR-1 staining similar to the negative control groups. Lower concentrations (3 µM—Figure 3c and 0.75 µM—Figure 3d) demonstrated moderate surface staining, whereas the positive control groups exhibited intense staining on both the cell surface and cytoplasm. These findings indicate that non-cytotoxic doses of dabigatran exhibit no statistically significant reduction in cell viability compared to control groups at lower concentrations cytotoxic and apoptotic effects on ADMSCs, while providing a controlled suppression of PAR-1 activity. Although PAR-1 is closely associated with cytoskeletal remodeling and cell migration, functional migration assays were not included in this study. Future studies incorporating wound healing or transwell migration assays would be valuable to determine whether the observed reduction in PAR-1 expression translates into altered MSC migratory capacity.

While our study is limited to an in vitro cell culture model and may not fully capture clinical outcomes, it is important to recognize that a commonly used anticoagulant could exert meaningful and potentially distinct effects on MSCs, the key mediators of tissue regeneration.

## 4. Materials and Methods

### 4.1. Materials

Dabigatran etexilate was obtained from Merc SA, Darmstadt, Germany (Cat-No: 211915-06-09); thrombin was obtained from Merc SA, Darmstadt, Germany (Cat-No: 9002-04-4); and PAR-1 (IF) was obtained from Thermo Fisher Scientific, Waltham, MA, USA (Cat-No: PA5-116040). DMEM/F12 (DMEM-12-HXA), FBS (Cat-No: FBS-16B), and trypsin-EDTA (Cat-No: TRY-3B) were obtained from Capricorn, Ebsdorfergrund, Germany.

### 4.2. Methods

#### 4.2.1. Cell Cultures

In this study, ADMSCs that were previously isolated from rat adipose tissue, then characterized and cryopreserved in our laboratory, were used in accordance with the approval of the Hacettepe University Local Animal Experiments Ethics Committee (Protocol No. 2013/04-04, date of approval: 18 February 2012, Monday). For each analysis, cells were seeded into 96-well plates at an initial density of 50,000 cells/mL with six replicates (n = 6).

Cells were incubated in Dulbecco’s Modified Eagle’s Medium (DMEM)/Ham’s F12 supplemented with 10% fetal bovine serum (FBS), 0.1 IU/mL thrombin, and five different concentrations of dabigatran etexilate 24 µM, 12 µM, 6 µM, 3 µM, and 0.75 µM at 37 °C in a humidified atmosphere containing 5% CO_2_ for 24 h. The negative control groups did not contain dabigatran or thrombin, whereas the positive control groups contained thrombin but not dabigatran. Thrombin concentration was selected based on previous studies demonstrating its effectiveness in inducing PAR-1 activation in vitro without causing excessive cytotoxicity [29,30]. Dabigatran concentrations and evaluation periods were selected based on our previous study [12]. Dabigatran etexilate, a prodrug form of dabigatran, was used in this study. Although it requires metabolic activation in vivo, previous in vitro studies have demonstrated that it can exert biological effects under cell culture conditions. The compound was prepared according to the manufacturer’s instructions and freshly diluted in culture medium prior to each experiment. The 24 h time point was selected based on our previous study and preliminary experiments, which indicated that this duration is sufficient to detect both cytotoxic and early apoptotic effects of dabigatran. However, we acknowledge that additional time points could provide further insight into the temporal dynamics of these effects.

#### 4.2.2. Assessment of Cell Viability and Morphology

Cell viability was assessed using the MTT assay as described previously [12,31,32]. and cell morphology was evaluated using acridine orange/propidium iodide (AO/PI) staining. After 24 h of incubation, the medium was removed, and AO/PI solution was added without fixation (*v*/*v* 1:1) and incubated for 20 s. Cells were washed with phosphate-buffered saline (PBS) and mounted using a PBS:glycerol (1:1) solution. Morphological changes were evaluated under an inverted fluorescence microscope and compared with control groups (Olympus IX70, Hachioji-shi, Tokyo, Japan). Viable cells emitted green fluorescence, whereas apoptotic and dead cells showed orange/red fluorescence.

#### 4.2.3. PAR-1 Immunofluorescence Staining

After 24 h of incubation, cells were washed with PBS and fixed with methanol for 10 min. Blocking serum was applied for 20 min, followed by incubation with primary anti-PAR-1 antibody for 60 min. Cells were then incubated with secondary antibody for 45 min, washed with PBS, and examined under a fluorescence microscope. Fluorescence images were acquired using an inverted fluorescence microscope (Olympus IX70, fluorescence attachment). Cells were visualized using a FITC filter (excitation ~498 nm, emission ~517 nm).

#### 4.2.4. Statistical Analysis

Statistical analyses were performed using SPSS version 23.0. Data were expressed as mean ± standard deviation. Normality was assessed using the Kolmogorov–Smirnov test. One-way ANOVA followed by LSD or Tamhane’s post hoc test was used for group comparisons. A *p* value < 0.05 was considered statistically significant.

## 5. Conclusions

The results of the present study demonstrate that dabigatran, at low doses, exerts tolerable cytotoxic and apoptotic effects while concurrently suppressing PAR-1 activity. Nonetheless, several limitations should be noted. The in vitro design precludes definitive conclusions regarding the behavior of PAR-1-inhibited ADMSCs in vivo. Moreover, the impact of dabigatran on other PAR-1-mediated functions, beyond its inhibitory effects and influence on coagulation, remains to be elucidated. Although limited by its in vitro design, these findings provide a foundation for future research on the impact of anticoagulants on MSC function and tissue regeneration, underscoring the need for in vivo validation.

## Figures and Tables

**Table 1:** MTT results of dabigatran at 24 h in each dilution compared to control groups.

Time (hours)	Dilutions	Mean	Standard Deviation	*p*
24	24 µM	0.045	0.018	0.000
	12 µM	0.124	0.006	0.457
	6 µM	0.126	0.008	0.273
	3 µM	0.121	0.007	0.687
	0.75 µM	0.124	0.006	0.400
	(-) Control	0.121	0.018	1
	(+) Control	0.112	0.007	1

Mean: Absorbance (OD), *p* values compared to control groups. In (-) control groups, medium do not contain dabigatran and thrombin, while (+) control groups contain thrombin, but not dabigatran.

**Table 2:** The ratio of apoptotic cells at 24 h in each dilution and control groups.

Time (hours)	Dilutions	Percent of Apoptotic Cells	Percent of Normal Cells
24	24 µM	100	-
	12 µM	9	91
	6 µM	7	93
	3 µM	6	94
	0.75 µM	6	94
	(-) Control	2	98
	(+) Control	5	95

In (-) control groups, medium do not contain dabigatran and thrombin, while (+) control groups contain thrombin, but not dabigatran.

**Table 3:** The ratio of PAR-1 (+) cells at 24 h in each dilution and control groups.

Time (hours)	Dilutions	Percent of PAR-1 (+) Cells
24	24 µM	-
	12 µM	30
	6 µM	25
	3 µM	60
	0.75 µM	65
	(-) Control	20
	(+) Control	75

In (-) control groups, medium do not contain dabigatran and thrombin, while (+) control groups contain thrombin, but not dabigatran.

## Data Availability

The raw data supporting the conclusions of this article will be made available by the authors on request.
